# *In Vitro* and *In Vivo* Evaluation of Polyherbal Formulation against Russell's Viper and Cobra Venom and Screening of Bioactive Components by Docking Studies

**DOI:** 10.1155/2013/781216

**Published:** 2013-02-28

**Authors:** G. Sakthivel, Amitabha Dey, Kh. Nongalleima, Murthy Chavali, R. S. Rimal Isaac, N. Surjit Singh, Lokesh Deb

**Affiliations:** ^1^Department of Nanotechnology, Noorul Islam Centre for Higher Education, Kumaracoil, Thuckalay, Tamilnadu 629180, India; ^2^Pharmacology Laboratory, Medicinal Plants & Horticultural Resources Division, Institute of Bioresources & Sustainable Development, Department of Biotechnology, Government of India, Takyelpat, Imphal, Manipur 795001, India

## Abstract

The present study emphasizes to reveal the antivenom activity of *Aristolochia bracteolata* Lam., *Tylophora indica* (Burm.f.) Merrill, and *Leucas aspera S.* which were evaluated against venoms of *Daboia russelli russelli* (Russell's viper) and *Naja naja* (Indian cobra). The aqueous extracts of leaves and roots of the above-mentioned plants and their polyherbal (1 : 1 : 1) formulation at a dose of 200 mg/kg showed protection against envenomed mice with LD_50_ doses of 0.44 mg/kg and 0.28 mg/kg against Russell's viper and cobra venom, respectively. In *in vitro* antioxidant activities sample extracts showed free radical scavenging effects in dose dependent manner. Computational drug design and docking studies were carried out to predict the neutralizing principles of type I phospholipase A_2_ (PLA_2_) from Indian common krait venom. This confirmed that aristolochic acid and leucasin can neutralize type I PLA_2_ enzyme. Results suggest that these plants could serve as a source of natural antioxidants and common antidote for snake bite. However, further studies are needed to identify the lead molecule responsible for antidote activity.

## 1. Introduction

Every year snake bites cause innumerable mortalities in India. Recent annual report estimates every year nearly 94,000 deaths globally and 15,000 deaths in India due to snake bites [1]. Snake venoms are heterogeneous mixture of organic and inorganic substances which is mainly used by the snake to immobilizae and to digest the prey. It is also used as a defense mechanism against their natural predators [2]. There are 3000 species of snakes known to science; in this 30% of snakes are venomous and considered dangerous to human [3]. Elapidae and Viperidae are two families of species which cause human mortality worldwide [4]. The Elapidae family contains the cobras and their relative species. All types of vipers come under the family Viperidae. Snake envenoming causes severe local tissue damage in the victim, such as hemorrhage and myonecrosis [2]. 

*Daboia russelli russelli* (Indian subspecies of Russell's viper) appears to be the commonest cause of fatal snake bite in Southern India [5]. Russell's viper venoms act predominantly on the haemostatic system, particularly on capillary endothelium which causes swelling within minutes of the bite [4]. In cobra bite local necrosis is the most common consequence and approximately half of the victims suffer local tissue necrosis, which is difficult to treat [6]. Phospholipase A_2_ (PLA_2_), neurotoxins, and cardiotoxins are the major classes of cobra venom polypeptides involved in the toxicity and pharmacology of bite [7]. PLA_2_ can be divided into three types based on its chemical structures. Elapidae and sea snakes from the genus *Hydrohis* venom contain type I PLA_2_. The venom of Viperidae and Crotalidae families contain type II PLA_2_. Venom of bees (*Apis mellifera*) and Gila monsters (*Heloderma*) contain type III PLA_2_ [8]. Many extensive studies have been done on this enzyme due to its pharmacological and physiopathological effects in living organisms. PLA_2_ activity upon cell membranes of specific tissues suggests an important role of these enzymes in venoms toxicity [9]. Antiserum, the only remedy for envenomation, may be associated with various reactions such as early anaphylactoid reaction, pyrogenic and late serum sickness, and several other manifestations [10]. In many cases the mortality of victim happens due to the wrong choice of antiserum because of misidentified snake species by the treating physicians which results in severe life-threatening envenoming to the victim [11]. In addition antiserum development in animals (like horse, goat, sheep, etc.) is time consuming, expensive, and requires ideal storage conditions [12]. In recent years, because of the costs as well as serious side effects of a number of modern drugs, attention has turned back to medicinal plants as drugs. It has been reported that about 64% of the total world population is using traditional medicine to satisfy their healthcare needs [13]. The neutralizing activity of plants against snake venom has long been recognized, but scientist paid attention to these medicinal plants only from the last 20 years [14]. Many Indian medicinal plants have been recommended by the traditional physicians for the treatment of snak ebite [15].

*Aristolochia bracteolata *Lam. (AB), *Tylophora indica* (Burm.f.) Merrill. (TI), and *Leucas aspera (S.) Labiate* (LA) are the major medicinal plants commonly found in India. AB belongs to the family Aristolochiaceae. Its roots and leaves are used as anthelminthic [16]. AB is used in traditional medicine as a gastric stimulant and in the treatment of cancer, lung inflammation, and dysentery [17]. TI commonly known as “Antmool” is an important medicinal plant traditionally used as a folk remedy in treatment of bronchial asthma, bronchitis, rheumatism, allergies, and inflammation. The roots and leaves contain 0.2 to 0.46% of therapeutically important alkaloids tylophorine which act as immunosuppressive, anti-inflammatory, antitumor stimulant of adrenal cortex and antiamoebic properties [18]. Leaves of LA are used for the treatment of respiratory tract disorders, edema, gastrointestinal disorders, general pain, and also as an antidote to poison. It plays a major role in Indian traditional medicine to treat psoriasis, chronic skin eruption, and chronic rheumatism [19]. The present study aims to examine the potential of polyherbal formulation of plant extracts to act as an antidote to neutralize snake venoms and screening of potent active herbal compounds by docking study.

## 2. Materials and Methods

### 2.1. Snakes Venom

The Indiancobra and Russell's viper venom was obtained from Irula Snake Catchers I.C.S. Ltd., Vadanemmeli village, Kancheepuram District, Tamilnadu, India and was preserved at 4°C. Before use, the venom was dissolved in normal physiological saline and centrifuged at 200 ×g for 10 min and the supernatant was used for anitvenom studies. Venom concentration was expressed in terms of dry weight.

### 2.2. Preparation of Plant(s) Extract

The *Aristolochia bracteolata *Lam. (AB), *Tylophora indica *(Burm.f.) Merrill. (TI), and *Leucas aspera* (S.) Labiate (LA) plants were identified and authenticated with the help of herbarium of S.T. Hindu college by Dr. B. Parthipan, Associate professor, Department of Botany and Research Center, S.T. Hindu College, Nagercoil, Kanyakumari District, Tamilnadu, India. Plants were collected from the foothill of Marunthuvazh malai (terminal of western gates) Nagercoil, Kanyakumari District, Tamilnadu and the details where noted in the field notebook according to field and herbarium techniques. The leaves and roots of the plants AB, TI, and LA were washed and homogenized. The shade-dried plant parts were brought to Pharmacology laboratory, IBSD, Imphal. The dried plants parts were powdered and subjected to soxhlet extraction (70 ± 10°C temp.) with the purified water as solvent in 1 : 4 (w/v) ratios for 18 hrs. The extracts were concentrated using rotary vacuum evaporator (40°C; pressure 70 ± 5 psi); the thick brown masses obtained were kept in vacuum desiccators for complete drying. The dried extracts were stored in air-tight container and kept in refrigerator (<10°C). The extracts were suspended in distilled water and used for pharmacological experiments.

### 2.3. Phytochemical Screening

Preliminary phytochemical screening was carried out according to the procedure given by Harborne [20], which revealed the presence of lipids, carbohydrates, flavonoids, and alkaloids in all the plant extracts.

### 2.4. Animals for Experimentations

Wistar albino rats (150–200 g) and Swiss albino mice (20–30 g) of either sex were provided by the institutional central animal house (IBSD, Imphal west, Manipur, India). The animals were acclimatized for one week under laboratory conditions. These were maintained under standard environmental condition (room temperature 27 ± 3°C, relative humidity 65 ± 10%, and 12-hour light/dark cycle). All the animals were fed with standard diet and water *ad libitum*. The litter in the cages was removed daily to ensure hygienic condition and maximum comfort for animals. Experiments were performed in accordance with the current guidelines of CPCSEA India. Ethical clearance for handling and experimentation on the animals was obtained from the Institutional Animals Ethical Committee (IAEC), IBSD, Imphal (Approval no. IBSD/IAEC/Inst./Ph.cology/3) prior to the commencement of the experimental works.

### 2.5. *In Vitro* Antisnake Venom Activity

#### 2.5.1. PLA_2_ Inhibition Activity

A mixture A mixture containing 1.2% egg yolk (dissolved in diethyl ether) as a source of lecithin, different concentration of aqueous extracts of AB, TI and LA (10 to 500 *μ*g) in 1 mL, 1 mL venom (200 *μ*g/mL) and 0.1 mL 5% CaCl_2_ in respective test tubes and the reaction tubes were spin until a homogeneous mixture was obtained. The reaction was allowed to stand under room temperature for 4 hrs. At the end 25 mL ethanol and 0.3 mL of cresol red (HiMedia, India) were added and titrated with ice cold 0.02 N methanolic NaOH. A blank was prepared by addition of ethanol, venom, and CaCl_2_ to the ether in same order and was titrated immediately. The hydrolysis capacity of PLA_2_ exhibited by reacting 200 *μ*g/mL of venom with lecithin, was considered as 100% phospholipase activity, and served as control [21].

#### 2.5.2. Inhibition of Adenosine-Diphosphate-(ADP-)Induced Platelet Aggregation

Platelet-rich plasma from healthy albino rats (*n* = 3) was obtained by centrifugation of their citrated blood at 190 ×g for 15 min. The reaction mixture contains 0.5 mL different concentration of aqueous extracts of AB, TI, and LA (10 to 500 *μ*g/mL), venom solution 0.5 mL (200 *μ*g/mL), and platelet-rich plasma 0.5 mL. Theis reaction mixture was maintained at 37°C and kept for 2 min with constant stirring; after this 0.5 mL of ADP solution (Sigma-aldrich) was added and incubated for 4 min in the dark and the absorbance was measured at 414 nm with Thermo Multiskan Spectrum [21]. The results were expressed as IC_50_ or inhibitory concentration 50 value:

(1) %  ADP  induced  platelet  aggregation =  (OD1−OD2)−(OD1−OD3)(OD1−OD2)  ×  100,

where OD1 = ADP + platelet, OD2 = ADP + venom + platelet, and OD3 = ADP + venom + platelet + plant  extract.

#### 2.5.3. Inhibition of Venom-Induced Hemolysis Assay

The hemolytic activity of aqueous extract of AB, TI, and LA was determined using the method of Oguiura et al. [22] and modified in our laboratory condition. Briefly, ethylene diamine tetra-acetic acid (EDTA) anticoagulated blood was collected from healthy Swiss albino mice, centrifuged at 300 ×g for 15 min, washed twice with PBS (pH 6.8), and diluted to 0.5% in phosphate buffer saline (PBS) with 10% fetal bovine serum, followed by dispensing 100 *μ*L into micro centrifuge tubes, to that different concentration of cobra and Russell's viper venom (10 *μ*L) was separately added in triplicate and incubated at 37°C for 2 hrs. All the tubes were centrifuged at 800 ×g for 10 min; the supernatants were transferred to 96-well plates and absorbance was measured at 405 nm for released hemoglobin. Control tubes 0 and 100% hemolysis consisting of red blood corpuscle (RBC) suspended in PBS and 1% Triton X-100, respectively. Inhibitor tubes consisting of 100 *μ*L of RBC suspended in PBS, 100 *μ*L of different concentration of test samples (aqueous extracts of AB, TI, and LA), and 10 *μ*L of venom at a concentration of 40 *μ*g. The results were expressed as IC_50_ or inhibitory concentration 50 value, that is, concentration of samples exhibited 50% inhibition of venom-induced hemolysis.

#### 2.5.4. Determination of Acute Toxicity (LD_50_) of Plant Extracts

The acute toxicity of aqueous extracts of AB, TI, and LA was determined in male albino mice maintained under standard conditions. The animals were fasted overnight prior to the experiment. Acute oral toxicity-Acute toxic class method (OCED Guideline no. 423, Annexure-2d) adopted by CPCSEA, Government of India was followed for acute toxicity studies. The behavioral abnormality, normal food and water intake, and mortality were observed after single oral administration of 2000 mg/kg b.w. of each test samples to their respective groups (*n* = 3) of animals. Common side effects such as mild diarrhoea, loss of weight, and depression of treated groups of animals were recorded within the 7 days of observation [23].

### 2.6. *In Vivo* Anti Snake Venom Activity

#### 2.6.1. Determination Median Lethal Dose (LD_50_) for Venom

For each venom, the lethal toxicity study was maintained under standard conditions and the animals were fasted overnight prior to the experiments. The median lethal toxicity of *Vipera russelli *and *Naja naja* venoms was performed by i.p. administration of different concentrations of venom dissolved in 0.2 mL of normal physiological saline to respective groups of male Swiss albino mice (*n* = 6). The median lethal dose (LD_50_) of venom was determined from the occurrence of death within 24 hrs of venom injections by probit analysis method [24].

#### 2.6.2. Neutralization Potential of Different Extracts and Polyherbal Formulation on Lethal Venom Effect

The overnight-fasted Swiss albino mice were divided into different treated groups (*n* = 5) and the venom-neutralizing potency of the aqueous extracts of AB, TI, and LA and polyherbal formulation (AB : TI : LA = 1 : 1 : 1) was evaluated. All the test groups of animals were envenomed with i.p. administration of LD_50_ (0.28 mg/kg b.w. for cobra venom and 0.44 mg/kg b.w. for Russell's viper venom) dose of venom suspension in physiological saline. After 5 minutes venom-challenged animals were administrated with the extracts at increasing doses of 150 mg/kg and 200 mg/kg b.w. (p.o.) and polyherbal formulation of 200 mg/kg b.w. (p.o.) to their respective test groups. The behavioral abnormality and mortality were observed after 1 hr of venom challenged followed by 48 hrs [25].

#### 2.6.3. Neutralization of Hemorrhagic Activity of Snake Venoms

The minimum hemorrhagic dose (MHD) was defined as the least dose of venom (*μ*g/b.w) which induces a hemorrhagic lesion of 10 mm in diameter when injected into rats intradermally after 24 hrs. Aliquots of venoms (100 *μ*L) in physiological saline or saline alone (control) were injected into the shaved dorsal skin of male Wister rats (150–200 g). In a single group five rats were used for each venom study. Aqueous extracts of AB, TI, and LA and polyherbal formulation (AB : TI : LA = 1 : 1 : 1) at 200 mg/kg p.o. were administered to their respective groups of animals just after 5 min of venom challenged [25]. 

### 2.7. *In Vitro* Antioxidant Activity

#### 2.7.1. DPPH Free Radical Scavenging Assay

The free radical scavenging activity of aqueous extracts of AB, TI, and LA was measured in terms of hydrogen donating or radical scavenging ability using the stable radical 2,2-diphenyl-1-picrylhydrazyl (DPPH) using the method of Jain et al. [26]. Solution of 0.1 mM DPPH (HiMedia, India) in ethanol was prepared and 1.0 mL of this solution was mixed with 3.0 mL of extract solution in water at different concentrations (10–100 *μ*g/mL). Thirty minutes later the absorbance was measured at 517 nm. Lower absorbance of the reaction mixture indicates higher free radical scavenging activity. Rutin (Ozone, India) was used as a standard. The results were expressed as IC_50_ or inhibitory concentration 50 value, that is, concentration of samples exhibited 50% inhibition of DPPH free radicals:

(2) $$\begin{array}{c} \%\;\text{Inhibition}=\left(\frac{A_{\text{blank}}-A_{\text{sample}}}{A_{\text{blank}}}\right)\times 100. \end{array}$$

#### 2.7.2. Superoxide Radical Scavenging Activity

The assay was based on the capacity of the aqueous extracts of AB, TI, and LA to inhibit blue formazon formation of scavenging the superoxide radicals generated in riboflavin-light-NBT (nitroblue tetrazolium) system. 100 *μ*L riboflavin solution, 200 *μ*L EDTA solution, 200 *μ*L ethanol, and 100 *μ*L NBT solution were mixed in a test tube and the reaction mixture was diluted up to 3 mL with phosphate buffer (pH 7.2). The absorbance of solution was measured at 560 nm using phosphate buffer as blank after illumination for 15 min. This was taken as control reading. Different concentration (10–100 *μ*g) of test samples in 100 *μ*L was mixed with 100 *μ*L riboflavin, 200 *μ*L EDTA, 200 *μ*L ethanol and 100 *μ*L NBT solution in the test tubes, and then the reaction mixtures were diluted to 3 mL with phosphate buffer. The absorbance of solution was measured after illumination for 15 min at 560 nm [27]. L-ascorbic acid was used as a reference control. Decrease in absorbance of the reaction mixture indicated increased superoxide anion scavenging activity. Percentage inhibition was calculated and this activity was expressed as an inhibition concentration 50 (IC_50_):

(3) $$\begin{array}{c} \%\;\text{Inhibition}=\left(\frac{{\text{OD}}_{\text{Control}}-{\text{OD}}_{\text{Test}}}{{\text{OD}}_{\text{Control}}}\right)\times 100. \end{array}$$

#### 2.7.3. Reducing Power Method

Different doses of aqueous extracts of AB, TI, and LA were mixed in 1 mL of distilled water so as to get 10 *μ*g, 20 *μ*g, 40 *μ*g, 80 *μ*g, and 100 *μ*g concentrations in separate test tubes and were mixed with phosphate buffer (2.5 mL, 0.2 M, pH 6.6) and potassium ferricyanide (2.5 mL, 1%). The mixture was incubated at 50°C for 20 min. A portion (2.5 mL) of trichloroacetic acid (10%) was added to the mixture, which was then centrifuged at 300 ×g for 10 min. The upper layer of the solution (2.5 mL) was mixed with distilled water (2.5 mL) and ferric chloride (0.5 mL, 0.1%), and the absorbance (OD) was measured at 700 nm. Rutin (Ozone, India) was used as a reference control. Increased absorbance of the reaction mixture indicates increase in reducing power. The % reducing power was calculated by using the formula [28]:

(4) $$\begin{array}{c} \%\;\text{reducing}\;\text{power}=\left(\frac{{\text{OD}}_{\text{Test}}-{\text{OD}}_{\text{Control}}}{{\text{OD}}_{\text{Test}}}\right)\times 100. \end{array}$$

### 2.8. Ligand Structure

**Figure 1:**
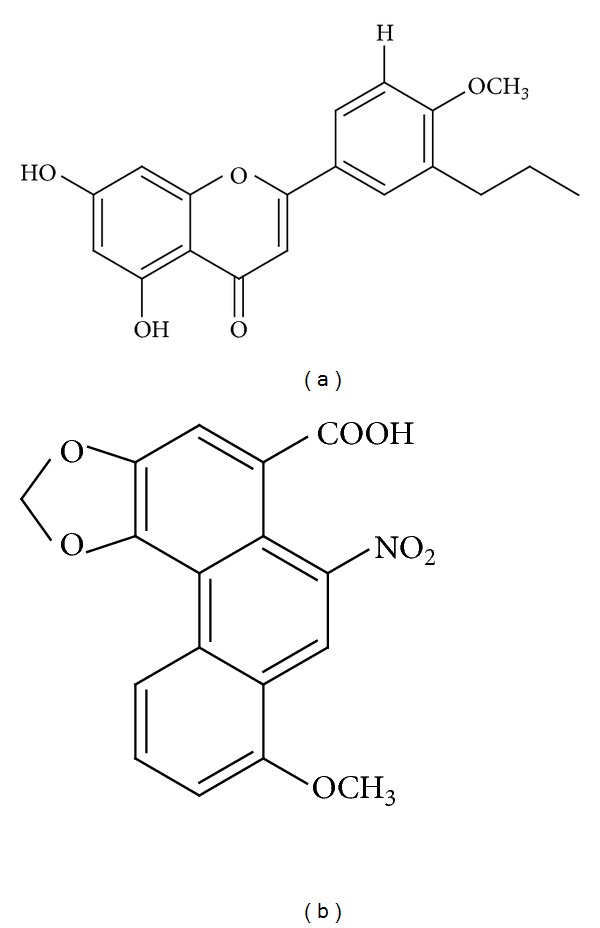
Structure of (a) leucasin isolated from LA, (b) aristolochic acids from AB.

The structure of Aristolochic Acids from AB and Leucasin from LA (Figure 1) were designed using the MedChem designer v1.0 based on Zhao et al and Meghashri et al. [29, 30] and the structures are saved in MDL (MOL format). This MOL-formatted chemical ligand structures were given as the input file for Accelrys Discovery Studio v2.5 and Glide v4.5 for energy minimization and subjected to subsequent docking by the Ligandfit method. All the properties of the compound where calculated for the validation of the ligand structure.

### 2.9. Protein Active Site Prediction

The potential binding sites (PBS) of proteins are those residues or atoms which bind to ligand directly on protein surface and were identified using MetaPocket 2.0 online server. In this, pocket sites are represented using the standard PDB file format. MetaPocket uses LIGSITE, PASS, Q-site finder, and SURFNET methods to identify pocket sites. All identified pockets are represented as a single probe with ranking score. To make the ranking scores comparable a Z-score is calculated separately for each site in different methods. Only the top most three pocket sites in each method are taken into further consideration. Probes within a certain distance threshold are grouped together as a cluster. Each cluster is ranked by a Z-scores, which is the sum of the Z-score of the pocket sites in a cluster [31].

### 2.10. Molecular Docking Protocols

Virtual screening and molecular docking studies are a series method in drug discovery. Most of the molecular docking algorithms assume the protein as rigid object which leads to poor correlation of the docking scores. There is no single docking algorithm or scoring function that can correctly predict the binding affinities of ligand in molecular interaction [32]. For these reason, in this study two docking programs such as LigandFit method in Discovery studio package and Glide were investigated to identify the interaction of ligand molecule in the active region of the protein and to predict the binding affinity between the ligand and the receptor protein molecule [33].

### 2.11. LigandFit/DS Docking

LigandFit method is an accurate orientation of the ligands into the protein active site region. The crystal structure determination of a basic PLA_2_ from common krait (*Bungarus caeruleus*) (PDB:1DPY) obtained from the RCSB Protein Data Bank (PDB) was used in computational study. At first, protein was prepared by removing all the water molecules and heteroatoms. Hydrogen atoms where added to the target protein structure using CHARMM force field as available in DS package V2.5 fallowed by minimization of added hydrogen using Smart Minimizer Protocol [34]. Throughout the docking process the top ten conformations were generated for each ligand after the energy minimization method based on steepest descent method and conjugate gradient method. Each of the saved conformation was evaluated and then ranked using the scoring function including Ligscore1, Ligscore2, PLP1, PLP2, JAIN, PMF, and LUDI. Visual inspections of receptor-ligand interaction were selected based on the Jain score for further evaluation [35]. The ligand which showed good hydrophobic and hydrogen bond interaction with active site I region of the receptor protein showed the possible character for neutralizing the activity of PLA_2_ from *Bungarus caeruleus* venom.

### 2.12. Glide

In the case of Glide v4.5, protein coordinates were preprocessed for docking using the Protein Preparation Wizard provided in the Schrodinger Maestro environment. Hydrogen atoms were added, and the bond orders were assigned after deleting the monomer B as well as water molecules. Assignment of protonation states was carried out followed by hydrogen bond optimization for hydroxyl group residues. The hydrogen atoms were then minimized with the OPLS2005 force field. Grid calculations were performed for the protein residues of the active sites coordinates. Initially Glide performs a complete systematic search of the conformational, orientational, and positional space of the docked ligand, eliminating unwanted conformations using scoring and followed by energy optimization. Finally the conformations are further refined via a Monte Carlo sampling of pose conformation. Predicting the binding affinity and rank-ordering ligands in database screens was implemented by modified and expanded version of the ChemScore18 scoring function. For our studies, three-dimensional crystal structure of a novel phospholipase A_2_ from Indian common krait was used (PDB:1DPY) with a resolution of 2.45 Å. Solvent molecules were deleted and bond order for crystal ligand and protein were adjusted and minimized up to 0.30 Å RMSD. Using standard precision (SP) mode of Glide software, docking studies were performed [34, 35].

## 3. Results

### 3.1. Determination of Acute Toxicity (ALD_50_)

The LD_50_ of Russell's viper and Indian cobra venom was established at 0.44 mg/kg and 0.28 mg/kg (20 g body weight) suspension in physiological saline on mice after intraperitoneal injection. All the test samples AB, LA and TI employed in acute toxicity study do not show any sign of abnormality and mortality applying dose of 2000 mg/kg in experimental animals for a period of 48 hrs initially and then after for a week of observation. Therefore 2000 mg/kg dose was considered as ALD_50_ cut off the dose (safe dose); accordingly 1/10 and less of that dose was selected for *in vivo* experiments.

### 3.2. *In Vitro* Anti Snake Venom Activity

The results of *in vitro* antivenom activities of aqueous extracts of AB, TI, and LA are shown in Table 1. For all *in vitro* assays aqueous extracts of AB was found to inhibit cobra venom effectively with IC_50_ values of 86 *μ*g, 74 *μ*g, and 386 *μ*g for PLA_2_ inhibition activity, inhibition of ADP-induced platelet aggregation, and inhibition of venom induced hemolysis assay, respectively. Whereas in case of Russell's viper venom LA showed highest activity with IC_50_ values of 134 *μ*g, 109 *μ*g, and 252 *μ*g, respectively.

### 3.3. *In Vivo* Anti Snake Venom Activity

The viper and cobra venom-induced lethality was neutralized by the aqueous extract of AB, TI, and LA in a dose-dependent manner, with the dose of 200 mg/kg (p.o) of LA showing more significant protection against Russell's viper venom. Whereas AB with the dose 200 mg/kg (p.o) protected against cobra venom. Approximately 200 mg/kg b.w. of polyherbal extract formulation in the ratio of (1 : 1 : 1 = AB : TI : LA) showed common antidote activity against both Russell's viper and Cobra venom (Tables 2 and 3). Venom-induced hemorrhagic activity was significantly inhibited by administration of the plant extracts (Table 4).

### 3.4. *In Vitro* Antioxidant Assay

**Figure 2:**
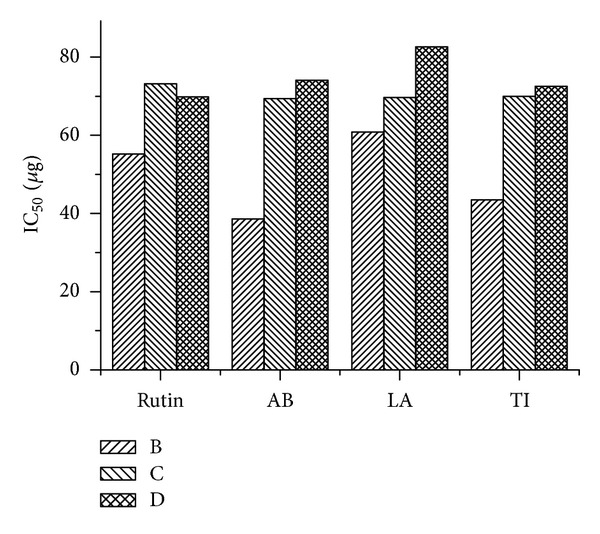
Histogram showing effect of Rutin (RU), *Aristolochia bracteolate* (AB), *Leucas aspera* (LA), and *Tylophora-indica* (TI) on DPPH* radicals (B), Reducing Power (C), and Superoxide scavenging activity (D) *in vitro*.

The reducing power activity, superoxide ion, and DPPH* free radical scavenging capacity of aqueous extracts of AB, LA, and TI were compared to standard Rutin (Tables 5, 6, and 7; Figure 2).

For the measurements of the reducing power ability, we investigated the Fe^3+^-Fe^2+^ transformation in the presence of different concentration of AB, TI, and LA, where a concentration dependent-reduction of ferric ions to ferrous ions was observed and their calculated EC_50_ values as 69.34 *μ*g, 69.65 *μ*g and 69.95 *μ*g which were found almost equal. 

In case of the nitroblue tetrazolium (NBT) superoxide radical system test samples AB, TI, and LA possesses concentration dependent inhibition of superoxide anions. The IC_50_ values of Rutin, AB, TI, and LA was calculated as 69.8 *μ*g, 74.05 *μ*g, 82.59 *μ*g and 72.5 *μ*g, respectively.

DPPH in presence of ethanol produces stable free radical and accepts hydrogen radical to become a stable diamagnetic molecule. Our study illustrates the hydrogen donating ability of AB, TI, and LA in various concentrations to scavenge DPPH free radicals and was compared with standard Rutin. The IC_50_ values of Rutin, AB, TI, and LA were calculated 55.24 *μ*g, 38.58 *μ*g, 60.86 *μ*g, and 43.47 *μ*g for DPPH radical scavenging effect, respectively.

### 3.5. Characterization of Protein Structure

**Figure 3:**
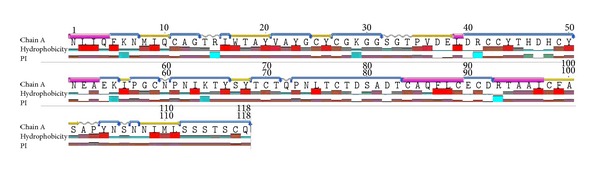
Geneious Basic annotation of the protein structure.

The crystal structure of a basic PLA_2_ from *Bungarus caeruleus* (PDB ID: 1DPY) was obtained from the RCSB Protein Data Bank (PDB) and used in computational study. Geneious Basic V5.4.2 is used for structure characterization to find which amino acids play important role in folding nature of the protein. The crystal structure of a basic PLA_2_ contains 118 amino acids with molecular weight 12.936 kDa and isoelectric point calculated as 4.94. Chain A “NLIQ-L-NEA-CAQFLC-TAAI” amino acid sequence showed Alpha Helix region & “FKN-CAG-T-VAYG-CGKGGS-DRCCYTHDHCY-EK-PGC-PNTKTY-TCT-PNLTCTDSADT-ECDR-YN-NN-STSCO” amino acid sequence showed turn region. Secondary structure of the phospholipase A_2_ contains 44.4% helix, 9.4% sheet, 5.1% turn, 33.3% coil, and 7.7% 3–10 helix region. Hydrophobicity and PI for the amino acids are also generated (Figure 3).

### 3.6. Protein Active Sites Prediction

**Figure 4:**
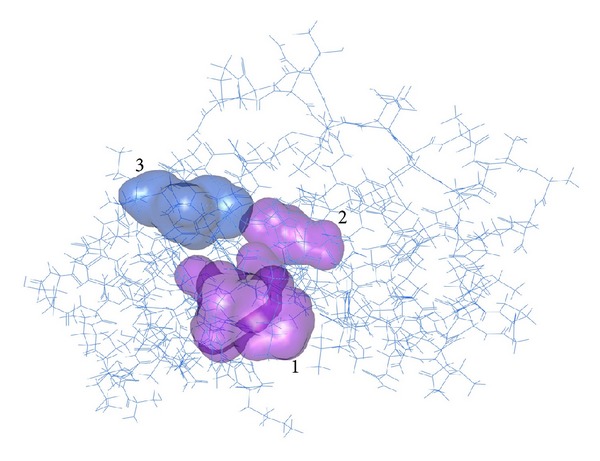
Protein structure is shown in wireframe model. Metapockets are shown in transparent solid surface form.

The potential binding sites (PBS) of proteins are those residues or atoms which bind to ligand directly on protein surface; they are near to the ligand binding sites. MetaPocket 2.0 online servers are used to find active site region of the protein. In this pocket sites are represented using the standard PDB file format. Potential 3 ligand binding sites were identified (Table 8; Figure 4).

### 3.7. Molecular Docking Analysis

**Figure 5:**
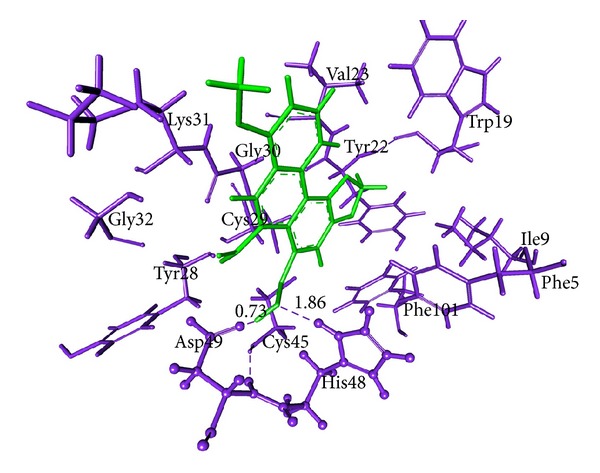
Aristolochic acids-PLA_2_ binding predicted using DS package v2.5. Protein residues (blue color) and ligand (green color) are represented by thin and sticks, respectively. Ligand binding regions are represented as ball and stick model. Hydrogen bonds are shown in blue dashed solid lines, respectively.

**Figure 6:**
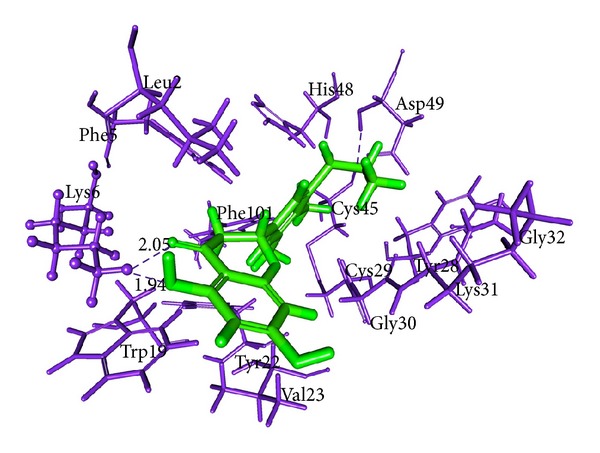
Leucasin-PLA_2_ binding predicted using DS package v2.5. Protein residues (blue color) and ligand (green color) are represented by thin and sticks, respectively. Ligand binding regions are represented as ball and stick model. Hydrogen bonds are shown in blue dashed solid lines, respectively.

**Figure 7:**
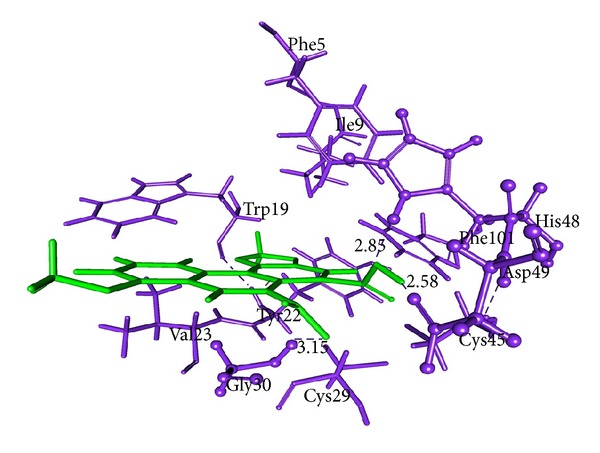
Glide v4.5 representation of Aristolochic Acids-PLA_2_ interaction. Protein residues (blue color) and ligand (green color) are represented by thin and sticks, respectively. Ligand binding regions are represented as ball and stick model. Hydrogen bonds are shown in blue dashed solid lines, respectively.

**Figure 8:**
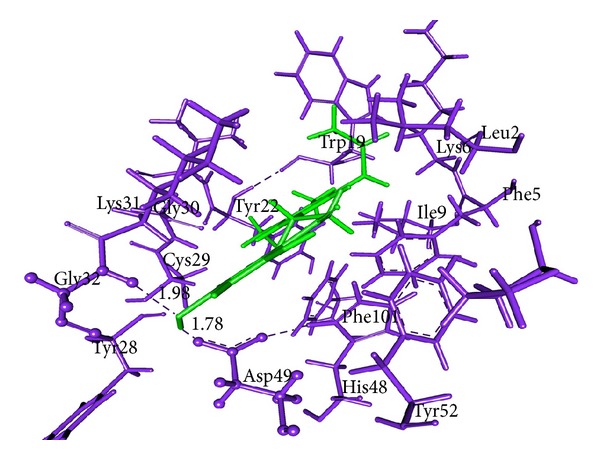
Glide v4.5 representation of Leucasin-PLA_2_ interaction. Protein residues (blue color) and ligand (green color) are represented by thin and sticks, respectively. Ligand binding regions are represented as ball and stick model. Hydrogen bonds are shown in blue dashed solid lines, respectively.

All dock runs were conducted using Discovery Studio v2.5 (DS) and Glide v4.5 to investigate the intermolecular interactions between the ligand and the target protein. The crystal structure determination of a Basic PLA_2_ from *Bungarus caeruleus* (PDB: 1DPY) obtained from the RCSB Protein Data Bank (PDB) was used in computational study. MedChem-designer-structured Aristolochic Acid and Leucasin were used as ligand molecule. Water molecules of crystallization were removed from the complex, and the protein was optimized for docking using the protein preparation and refinement utility provided by Discovery Studio v2.5 and Protein Preparation Wizard in Glide. Atomic charges were assigned according to the CHARRM force field and IPLH2005. Ligands Aristolochic Acid and Leucasin were prepared by the method discussed in DS package and Glide. Ligandfit method in DS v2.5 which generates 10 pose of ligand molecules docked to the receptor protein active site region 1. In that best docked pose of the ligand molecules were considered according to the docking score obtained from DS Ligandfit method (Figures 5 and 6; Tables 9 and 11). Glide v4.5 predicted two modes of interactions between ligand and PLA2 by means of hydrogen bond (Figures 7 and 8; Tables 10 and 12).

### 3.8. Methodology of Ligand-Protein Interaction

aristolochic acid shows interaction with Active site I region of PLA_2_ from *Bungarus caeruleus* by means of hydrogen bond. DS package and Glide were used to study the way of ligand-protein interaction. By our computational study we found that H33 of the Aristolochic acid form hydrogen bond with the receptor protein at ASP49 and another hydrogen bond occur between ASP49 with CYS45. The interaction between Leucasin and Active site I region of PLA_2_ happens by means of H-bond between ligand atom O18, O17 with the Lys6 of PLA_2_ by Ligandfit method in DS package (Figures 5 and 6). Glide result shown the hydrogen bond interaction occur between the H33 of the Aristolochic Acids and Lys31 from PLA_2_ and O7, H3 of Leucasin with Asp49, Gly32 (Figures 7 and 8). From these we conclude the mode of interaction of ligand molecule with the receptor protein by forming hydrogen bond which clearly showed the possibility that drug can neutralize common krait venom. 

## 4. Discussion

AB, TI, and LA are very common Indian medicinal plants that are used widely in Ayurvedic preparations to treat many diseases. Due to numerous therapeutic properties, these plants are used as an alternative medicine. The present work aimed to study the antidote activity of AB, TI, and LA against snake venom.

From the present study, the aqueous extracts of AB, TI, and LA were found to be neutralize the venom of Russell's viper and Cobra. Aqueous extract of AB showed 80% inhibition of lethality effect caused by Russell's viper and cobra venom. LA showed 60% inhibition of venom lethality effect of cobra and 100% in case of viper venom during 48 hours of observation. For cobra venom aqueous extracts of TI showed 60% inhibition of venom lethality up to 6 hours and 60% against Russell's viper venom up to 12 hrs. From the above results it is clearly understood that TI acts as a first aid in slowing down the time interval for venom spreading in the body. The polyherbal formulation of AB, TI, and LA (1 : 1 : 1) shows promising venom-neutralizing activity against cobra and viper venom.

PLA_2_ is commonly found in the venom of Viperidae, Hydrophiidae, and Elapidae family [8]. Neutralizing effect of the PLA_2_ activity is the common character of herbal drug which can act as the antidote for most types of snake venom. PLA_2_ type present in viper and cobra venom might release a large amount of platelet-activating factor in addition to histamine and other anaphylactic mediators from mast cells [24]. In the present study the protection of the mast cells against the degranulation effect of cobra and viper venom by the aqueous extract of AB, TI, and LA was studied. In this TI showed high phospholipase inhibition activity on cobra and viper venom by preventing the release of platelet-activating factor and histamine from mast cells followed by LA and AB. 

Local necrosis and hemorrhagic lesions were shown in some snake envenomation [25]. Hemorrhaging damages the vascular endothelium which leads to bleeding from vital organs and the victim will die due to excessive blood loss [36]. The aqueous extract of AB, TI, and LA shows significant reduction in viper and cobra venom-induced hemorrhagic damage. This inhibition activity of PLA_2_, ADP-induced platelet aggregation shows promising antivenom character of the polyherbal drugs since WHO report states that antivenom drugs should have capacity to neutralize venom lethality and haemorrhagic effects [24].

*In vitro *antioxidant DPPH free radical scavenging, superoxide radical scavenging, and reducing power activity of AB, TI, and LA were studied. Antioxidant properties of the plants play important role in neutralizing the snake venom [15, 21]. It was evident from our study that aqueous extract of AB, TI and LA showed dose dependent antioxidant activities. This result suggests the possible snake venom neutralizing activity due to free radical scavenging and antioxidant activity which may be due to the presence of phytoflavonoids in the plants which boost the antidote properties against snake venom. It was argued that disturbances in the second messenger and regulatory activity of free radicals may also contribute significantly to the various inflammatory processes.

It is evident from our study that aqueous extract of AB, TI, and LA showed dose-dependent antioxidant activities. This result suggests the possible snake venom neutralizing activity due to free radical scavenging, and antioxidant activity which may be due to the presence of phytoflavonoids in the plants which boost the antidote properties against snake venom.

*In vitro* studies showed that AB, TI and LA can induce PLA_2_ of cobra and Russell's viper venom. In order to know the neutralizing effect of the plants against PLA_2_ from *Bungarus caeruleus* venom computational docking methods were followed to screen the potent compound which may be the source of venom lethality and make a conclusion on the mode of interaction of herbal drug. The active site region for the enzyme PLA_2_ type I from *Bungarus caeruleus* venom (PDB:1DPY) is identified based on the bound inhibitor pocket region in the crystal structure. DS package and Glide docking score along with the binding orientation and hydrogen bond interaction were considered as the primary components to choose the best ligand-receptor interaction. In our study we focus on screening the possible potent compounds present in our plants against PLA_2_ from the venom of *Bungarus caeruleus* by computational docking study. Initially, based on several reviews we identified four different secondary metabolites from AB, TI, and LA which were designed using MedChem designer v1.0. In these four compounds Lecuasin from LA and Aristolochic acid from AB showed positive docking score for type I PLA_2_ of *Bungarus caeruleus* venom. Characterization study of the *Bungarus caeruleus* venom PLA_2_ structure showed that different amino acids play significant role in the formation of functionally important structure. Secondary structure of PLA_2_ contains 44.4% helix, 9.4% sheet, 5.1% turn, 33.3% coil, and 7.7% 3–10 helix region. The ligand active targets were present mostly in the coil region of PLA_2_ such as Arg49, His48 and Lys6. The potential ligand binding sites were examined for PLA_2_ and generated the binding sites I, II and III. H33 from aristolochic acid and Lecuasin O17, O18 showed the interaction with the Asp49 and Lys6 in binding site region I of PLA_2_ by means of forming hydrogen bond using Ligandfit method in DS package with the docking score 48.109 and 50.76. Glide docking parameter showed H33, O2 of aristolochic acid and H15, O4 of Lecuasin interacts with Lys31, His48, and Asp49, Gly32 in active region I of the PLA_2_ with the docking score −7.051, −6.585 and −6.852, −7.966. It is reported that type I PLA_2_ from snake venom catalyzes the hydrolysis of 2-acyl ester bond of 3-sn-phospholipids producing fatty acids and lysophospholipids. The ca^2+^ ion, an essential cofactor, and Asp49 are required for catalysis activity. These catalytic activities upon cell membrane of specific tissues suggest an important role of PLA_2_ in venom toxicity and cell death [9]. Our study suggests the neutralizing activity of aristolochic acid by means of forming H-bond interaction with the Asp49 which results in the alternation of the function, leading to blocking the catalytic activity of Ca^2+^ cofactor in PLA_2_ and prevent the cell death. In Glide docking study showed the H-bond interaction of O20 from aristolochic acid with the His48 of PLA_2_ which results in the reduction of His48 enzymatic and toxic activity. Hence His48 involved with the catalysis through its N-1 group oriented towards the solvent and made toxic effect [9]. His48 is supported by hydrogen bonds from Tyr52 to the side chain of opposite helix (Asp99) with close coupling of Asp49 and defines the active site geometry of type I PLA_2_ enzyme. There is an other statement as aristolochic acid failed to neutralize the toxic properties of type II PLA_2_ from russell's viper venom but in circular dichroism studies showed that binding of aristolochic acid alters the secondary structure of type II PLA_2_ from russell's viper; such a structural change may lead to lesser degrees of hemolysis and edema-inducing activity of this enzyme. In addition aristolochic acid is a competitive inhibitor of type I PLA_2_ and showed noncompetitive inhibition in the case of type II PLA_2_ from russell's viper venom [37, 38]. Type II PLA_2_ venom enzyme differs from type I PLA_2_ by having extended C-terminal tail [39]. This leads to differance in their pharmacological sites of the enzyme. 

In case of leucasin, it directly binds with PLA_2_ by means of forming H-bond between O18 and O17 of leucasin with Lys6 residues of PLA_2_. Lys6 residues are found in PLA_2_ of all Elapidae venom. It is reported that Lys6 plays an indirect role in the Ca^2+^ cofactor for catalyst activity of the PLA_2_ in snake venom by means of intermolecular H-bond with Ca^2+^catalytic active site residues [39]. Modification in the Lys6 might distort the binding ability of PLA_2_ for substrate and cause a drastic loss in enzymatic activity [40]. Leucasin atom O18 and atom O17 formed H-bond with the Lys6 which block the Ca^2+^ catalytic activity of His48 and Asp49, Since there is intermolecular H-bond between the Lys6, His48, Asp49, Lys2, Trp19 and Tyr22 which connects the active site residues to the N-terminal *α*-helix segment which promotes the Ca^2+^ catalytic activity. Our computational study shows that the screened potent secondary metabolites can also neutralize type I PLA_2_ enzyme from *Bungarus caeruleus* venom and these finding suggest that our selected plant can act as the source of common antidote for snake venom. 

## 5. Conclusion

Present crisis of dependable and safe antivenom supply in the developing countries reflects a global loss of momentum in anti-venom research, development, and financing. But the outcome of the present study is encouraging for research on antivenom from natural resources, where aqueous extracts of *Aristolochia bracteolata*, *Tylophora indica,* and *Leucas aspera* exhibited neutralizing effects against Russell's viper and Cobra venom. Further study on isolation of active phytoconstituents and their antisnake venom property could lead to the development of a new natural antidote for snake envenoming.

## Figures and Tables

**Table 1:** *In vitro* antisnake venom activity of aqueous extracts of AB, TI, and LA.

	IC_50_ value (*μ*g) of inhibition of					
Sample	Effect on platelets		PhospholipaseA_2_		Hemolysis	
	Viper venom	Cobra venom	Viper venom	Cobra venom	Viper venom	Cobra venom
AB	260 ± 0.062	86 ± 0.031	133 ± 0.029	74 ± 0.031	476 ± 0.032	386 ± 0.056
TI	469 ± 0.014	347 ± 0.012	139 ± 0.069	217 ± 0.089	724 ± 0.039	627 ± 0.029
LA	134 ± 0.087	122 ± 0.050	109 ± 0.031	57 ± 0.067	252 ± 0.041	718 ± 0.032

All values are expressed as a Mean ± SEM (*n* = 3).

**Table 2:** Effect of the aqueous extract of  AB, TI, and LA on the lethality of Naja Naja venom.

Group (*n* = 5)	*Naja naja* venom (mg/kg)	% survival 6 Hrs	% survival 12 Hrs	% survival 48 Hrs
Control (0.5 mL saline p.o.)	0.28	—	—	—
AB (200 mg/kg p.o.)	0.28	100	100	80
TI (200 mg/kg p.o.)	0.28	60	—	—
LA (200 mg/kg p.o.)	0.28	100	80	60
Polyherbal formulation (200 mg/kg p.o.)	0.28	100	100	60

Results were expressed as % survival of six mice in each group used under experiment.

**Table 3:** Effect of  the aqueous extract of  AB, TI, and LA on the lethality of Russell's viper venom.

Group (*n* = 5)	*Vipera russelli* venom (mg/kg)	% survival 6 Hrs	% survival 12 Hrs	% survival 48 Hrs
Control (0.5 mL saline p.o.)	0.44	—	—	—
AB (200 mg/kg p.o.)	0.44	100	100	80
TI (200 mg/kg p.o.)	0.44	80	60	—
LA (200 mg/kg p.o.)	0.44	100	100	100
Polyherbal formulation (200 mg/kg p.o.)	0.44	100	100	80

Results were expressed as % survival of six mice in each group used under experiment.

**Table 4:** Effect of the aqueous extract of  AB, TI, and LA on venom-induced hemorrhage effects.

Group (*n* = 5)	Venom-induced hemorrhage (MHD mean ± S.E.M)	
	*Vipera russelli* venom	*Naja naja* venom
Control (0.5 mL saline p.o.)	10.32 ± 0.236	10.03 ± 0.320
AB (200 mg/kg p.o.)	7.27 ± 0.327**	4.32 ± 0.411**
TI (200 mg/kg p.o.)	8.77 ± 0.421*	7.49 ± 0.231**
LA (200 mg/kg p.o.)	4.96 ± 0.330**	6.06 ± 0.332**
Polyherbal formulation (200 mg/kg p.o.)	5.42 ± 0.330**	6.38 ± 0.220**

All values are expressed as a mean ± SEM, *n* = 6. One way analysis of variance (ANOVA) followed by multiple comparison Dunnett's test. The minimum value of  *P* < 0.05 was considered significant. *P < 0.05, **P < 0.01,  compared to control group.

**Table 5:** Effect of  AB, TI, and LA on DPPH* radicals in *in* *vitro*.

Sl Number	Sample	IC_50_ value (*μ*g)
1	Rutin (reference Standard)	55.24
2	Aqueous extract of AB	38.58
3	Aqueous extract of TI	43.47
4	Aqueous extract of LA	60.86

The results were expressed asIC_50_(inhibitory concentration 50) value, that is, concentration of samples exhibited 50% inhibition of DPPH radicals.

**Table 6:** Effect of  AB, TI, and LA on reducing power.

SL Number	Sample	IC_50_ value (*μ*g)
1	Rutin (Reference Standard)	73.2
2	Aqueous extract of AB	69.34
3	Aqueous extract of TI	69.95
4	Aqueous extract of LA	69.65

The results were expressed as EC_50_ (effective concentration 50) value, that is, concentration of samples exhibited 50% reducing power activity.

**Table 7:** Effect of  AB, TI, and LA on superoxide radical scavenging activity.

SL Number	Sample	IC_50_ value (*μ*g)
1	Rutin (reference standard)	69.80
2	Aqueous extract of AB	74.05
3	Aqueous extract of TI	72.50
4	Aqueous extract of LA	82.59

The results were expressed as IC_50_ (inhibitory concentration 50) value, that is, concentration of samples exhibited 50% inhibition of superoxide radicals.

**Table 8:** The potential 3 ligand binding sites of the crystal structure determination of a basic PLA_2_ from common krait (*Bungarus caeruleus*).

	RN CI SN	RN CI SN	RN CI SN	RN CI SN	RN CI SN
Binding site ID: I					

RESI	TYR_A^*∧*^ 52^*∧*^	ASN_A^*∧*^ 53^*∧*^	GLU_A^*∧*^ 56^*∧*^	PRO_A^*∧*^ 63^*∧*^	ASP_A^*∧*^ 49^*∧*^
RESI	ASN_A^*∧*^ 64^*∧*^	ASN_A^*∧*^ 62^*∧*^	HIS_A^*∧*^ 50^*∧*^	CYS_A^*∧*^ 51^*∧*^	HIS_A^*∧*^ 48^*∧*^
RESI	LEU_A^*∧*^ 2^*∧*^	PHE_A^*∧*^ 5^*∧*^	TYR_A^*∧*^ 46^*∧*^	ALA_A^*∧*^ 97^*∧*^	PHE_A^*∧*^ 101^*∧*^
RESI	LYS_A^*∧*^ 6^*∧*^	ILE_A^*∧*^ 9^*∧*^	TRP_A^*∧*^ 19^*∧*^	TYR_A^*∧*^ 28^*∧*^	CYS_A^*∧*^ 45^*∧*^
RESI	CYS_A^*∧*^ 44^*∧*^	GLY_A^*∧*^ 32^*∧*^	LYS_A^*∧*^ 31^*∧*^	GLY_A^*∧*^ 30^*∧*^	CYS_A^*∧*^ 29^*∧*^
RESI	TYR_A^*∧*^ 22^*∧*^	GLY_A^*∧*^ 33^*∧*^	VAL_A^*∧*^ 23^*∧*^	THR_A^*∧*^ 20^*∧*^	ALA_A^*∧*^ 24^*∧*^
RESI	TYR_A^*∧*^ 25^*∧*^	SER_A^*∧*^ 34^*∧*^			

Binding site ID: II					

RESI	TYR_A^*∧*^ 52^*∧*^				

Binding site ID: III					

RESI	GLU_A^*∧*^ 40^*∧*^	ARG_A^*∧*^ 43^*∧*^	CYS_A^*∧*^ 44^*∧*^	CYS_A^*∧*^ 100^*∧*^	ALA_A^*∧*^ 104^*∧*^
RESI	SER_A^*∧*^ 103^*∧*^	THR_A^*∧*^ 47^*∧*^	HIS_A^*∧*^ 48^*∧*^	THR_A^*∧*^ 96^*∧*^	ALA_A^*∧*^ 97^*∧*^
RESI	CYS_A^*∧*^ 51^*∧*^	GLU_A^*∧*^ 92^*∧*^	CYS_A^*∧*^ 93^*∧*^	CYS_A^*∧*^ 45^*∧*^	

RESI: Residue line indicator, RN: Residue name, CI: Chain indicator, SN: Sequence number.

**Table 9:** Discovery Studio docking score of aristolochic acids—PLA_2_ binding. Docking process top ten conformations were generated for each ligand after the energy minimization method based on steepest descent method and conjugate gradient method. Each of the saved conformation was evaluated and then ranked using the dock score. Ligand pose 1 ranged as best pose with dock score and Jain value.

Lig pose	Lig score 1	Lig score 2	-PLP1	-PLP2	Jain	-PMF	Dock score	MWT	Lig.INE
1	2.87	4.01	65.73	56.18	−0.01	43.61	48.109	341.28	17.054
2	2.65	3.84	45.94	38.92	−0.67	56.46	46.988	341.28	11.694
3	2.25	3.58	62.88	52.65	−0.14	42.59	46.701	341.28	14.191
4	2.63	3.87	53.16	49.23	−0.46	60.07	46.696	341.28	11.898
5	2.63	3.87	53.16	49.23	−0.46	60.07	46.696	341.28	11.898
6	2.05	3.7	46.96	40.16	−1.02	57.5	46.496	341.28	11.68
7	2.33	3.63	59.87	50.33	−0.12	40.09	46.346	341.28	17.017
8	2.85	3.99	51.03	43.63	−0.87	61.62	46.826	341.28	13.07
9	2.57	3.77	41.79	38.24	−1.38	72.98	45.316	341.28	11.037
10	3.96	4.26	65.3	53.94	0.02	38.86	45.251	341.28	13.226

**Table 10:** Glide docking score for aristolochic acids—PLA_2_.  Hydrogen bond distance where measured in Å. Binding affinity and rank ordering were measured based on ChemScore18 scoring function.

Ligand interaction	Interaction	Distance (Å)	Dock score	Glide energy
Interaction 1	O–H*⋯*O(Gly30)	3.15	−7.051	−50.31
Interaction 2	O–H*⋯*O(Asp49)	2.58		
	(His48)N–H*⋯*O	2.85	−6.585	−52.521

**Table 11:** Discovery Studio docking score of Leucasin—PLA_2_ binding. Docking process top ten conformations were generated for each ligand after the energy minimization method based on steepest descent method and conjugate gradient method. Each of the saved conformation was evaluated and then ranked using the dock score. Ligand pose 1 ranged as best pose with dock score and jain value.

Lig pose	Lig score 1	Lig score 2	-PLP1	-PLP2	Jain	-PMF	Dock score	MWT	Lig.INE
1	2.27	3.76	48.71	49.93	−0.55	56.53	50.76	326.39	3.892
2	3.2	4.43	52.4	56.57	−0.08	77.62	48.765	326.39	5.913
3	2.03	3.68	41.8	40.89	−0.66	54.39	48.712	326.39	4.148
4	2.06	3.78	48.65	46.97	−1.07	56.7	48.674	326.39	5.113
5	2.83	3.58	48.66	53.82	−0.063	72.98	48.379	326.39	3.881
6	3.34	4.56	54.43	58.64	0.2	68.12	48.333	326.39	4.011
7	2.04	4.14	49.8	52.73	−0.99	77.05	48.293	326.39	3.645
8	2.36	3.8	39.58	39.05	−0.95	61.75	48.057	326.39	3.016
9	2.93	4.82	61.01	63.18	2.21	90.9	47.778	326.39	2.791
10	2.23	3.6	47.68	47.58	−1.07	56.3	47.326	326.39	4.69

**Table 12:** Glide docking score for Leucasin—PLA_2_. Hydrogen bond distances where measured in Å. Binding affinity and rank ordering were measured based on ChemScore18 scoring function.

Ligand interaction	Interaction	Distance (Å)	Dock score	Glide energy
Interaction 1	(Asp49)O*⋯*H	1.787	−7.966	−39.42
Interaction 2	(Gly32)H*⋯*O	1.998	−6.852	−36.55
